# Specific immunoglobulin G4 correlates with Th2 cytokine reduction in patients with allergic asthma treated by Dermatophagoides pteronyssinus subcutaneous immunotherapy

**DOI:** 10.1016/j.waojou.2022.100715

**Published:** 2023-01-26

**Authors:** Qiujuan Su, Nina Ren, Mulin Feng, Xueni Zeng, Yan Dong, Mo Xian, Xu Shi, Tian Luo, Gang Liu, Jing Li

**Affiliations:** a Department of Allergy and Clinical Immunology, Guangzhou Institute of Respiratory Health, State Key Laboratory of Respiratory Disease, The First Affiliated Hospital of Guangzhou Medical University, Guangzhou, China; b Yangjiang Key Laboratory of Respiratory Disease, Department of Respiratory Medicine, People's Hospital of Yangjiang, Yangjiang, China; c Department of Respiratory Medicine, Affiliated Hospital of Guangdong Medical University, Zhanjiang, China; d Clinical Research Center, Affiliated Hospital of Guangdong Medical University, Zhanjiang, China

**Keywords:** Allergen specific immunotherapy, Asthma, Regulatory T cells, Rhinitis, T helper cells

## Abstract

**Background:**

The modulations of lymphocyte subsets and cytokine production due to subcutaneous allergen immunotherapy (SCIT) are not fully clarified.

**Objective:**

We investigated the changes in T-lymphocyte subsets and serum *Dermatophagoides pteronyssinus*-specific immunoglobulin G4 (Der-p sIgG4), as well as cytokine production during Der-p SCIT, in patients with allergic asthma.

**Methods:**

This study involved 20 patients with allergic asthma who were receiving 156-week Der-p SCIT and 20 patients without SCIT (non-SCIT). We measured symptom and medication scores (SMS), serum Der-p sIgG4 levels, CD4^+^CD25^+^Foxp3^+^ T regulatory (Treg), CD4^+^IL-4^−^IFN-γ^+^ T-helper (Th) 1, and CD4^+^IL-4^+^IFN-γ^−^ Th2 lymphocyte percentages in peripheral blood mononuclear cells (PBMCs) with/without Der-p extract stimulation at weeks 0, 4, 12, 16, 52, 104, and 156. Cytokine release inhibition assays were performed by incubation with serum from SCIT and non-SCIT patients, Der-p allergen, and PBMCs. Levels of interleukin (IL)-4, IL-5, IL-10, IL-13, IL-17, interferon (IFN)-γ, tumor necrosis factor (TNF)-α, and transforming growth factor (TGF)-β1 were evaluated in supernatant.

**Results:**

We found that SCIT patients had significantly lower SMS after week 52. Der-p sIgG4 levels in SCIT patients significantly increased at week 16 compared with non-SCIT subjects. CD4^+^IL-4^+^IFN-γ^−^ Th2% in SCIT patients showed a significant decrease from weeks 104–156 compared with week 0, while no change was observed in CD4^+^CD25^+^Foxp3^+^ Treg and CD4^+^IL-4^−^IFN-γ^+^ Th1 percentages. IL-5, IL-13, IL-4, IL-17, and TNF-α levels in supernatant of PBMCs cultured with serum of SCIT patients after 16 weeks showed significant lower levels compared with non-SCIT patients, and showed significant reverse associations with Der-p sIgG4 levels.

**Conclusion:**

SCIT induced Dep-p sIgG4 may be involved in downregulating Th2 cytokine production in Der-p allergic asthma patients.

## Introduction

Allergen immunotherapy (AIT) is an effective treatment for allergic rhinitis and allergic asthma in terms of reducing symptom score and medication requirements, thereby improving quality of life, changing the course of allergic disease, and inducing allergen-specific immune tolerance.^1^ The mechanisms associated with immunotherapy involve changes in the humoral immune response and cellular reactions.^2^ Cellular changes include generation of allergen-specific T regulatory (reg) and Breg cells, and suppression of allergen-specific T-helper (Th) 2 cells.^3^ Tregs have been shown to govern peripheral self-tolerance in experimental animals and humans, suppress allergic responses to aeroallergens such as house dust mite and grass pollen, and shift Th2 to Th1, thereby reducing the release of Th2-type cytokines such as interleukin (IL)-4, IL-5, and IL-13. Treg-secreted IL-10 and transforming growth factor-β (TGF-β) switch allergen-specific B-cells to produce IgG4 instead of allergen-specific immunoglobulin E (sIgE).^4^ Our previous and other studies have found that AIT induces substantial production of allergen-specific immunoglobulin G4 (sIgG4) antibodies.^5^, ^6^ Specific IgG4 antibodies are thought to be responsible for competing with sIgE to form allergen-IgE complexes that inhibit the binding of sIgE to receptor-expressing effector cells^7^, ^8^, ^9^ and further reduce cytokine release. Although these observations contribute to clarifying how AIT promotes the induction of allergen tolerance, there are still unclear aspects. Namely, details of immunosuppression factors leading to reduction in cytokine release through modulating the *reactivity* or the *sensitivity* of effector cells during the long-term AIT are not clear. In this study, we investigated the relationship between IgG4 antibodies and Th2 cytokine release to demonstrate weather house dust mite subcutaneous allergen immunotherapy (SCIT) reduce Th2 cytokine production is associated with induction of IgG4 in patients with allergic rhinitis and/or asthma.

## Materials and methods

### Study design and population

**Fig. 1:**
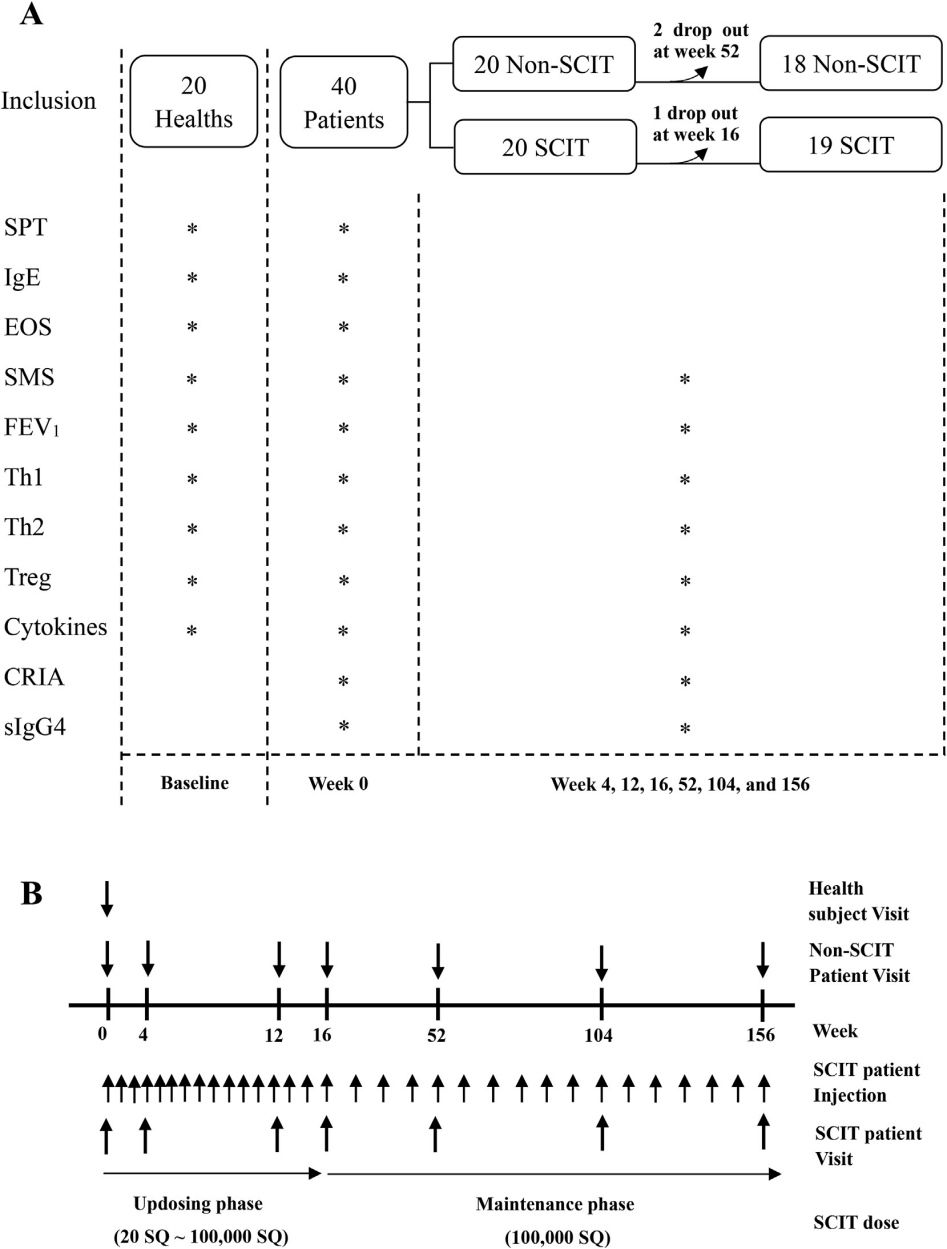
**Flowchart of the study design, clinical visits, and therapy schedule**. (A) Study design and biological parameters measured in the different groups. (B) Dosing schedule of SCIT injection and visits for clinical evaluation and blood sampling in SCIT and non-SCIT groups. ∗ performed; SCIT, subcutaneous allergen immunotherapy; SPT, skin prick test; IgE, immunoglobulin E; EOS, peripheral eosinophil counts; SMS, symptom and medication score; FEV1, forced expiratory volume at 1 s; PBMC, peripheral blood mononuclear cells; Th, T helper cell; Treg, regulatory T cell; CRIA, cytokine release inhibition assay; sIgG4, specific immunoglobulin G4.

The study included 60 subjects (37 males and 23 females, 5–58 years of age), of which 20 patients received *Dermatophagoides pteronyssinus* (Der-p) SCIT, 20 patients received standardized asthma and rhinitis medications (non-SCIT), and 20 healthy subjects were taken as baseline control (Fig. 1A, Table 1). The patients were predominantly sensitized to house dust mites and those having concomitant sensitization to other allergens with serum sIgE greater than or equal to 0.70 kU/L were excluded from the study. All patients came from the allergy and clinical immunology department, fulfilled the Allergic Rhinitis and its Impact on Asthma (ARIA) guidelines for allergic rhinitis and/or Global Initiative for Asthma (GINA) guidelines for mild-to-moderate asthma,^10^, ^11^ and had a positive skin prick test (SPT) and sIgE to Der-p. Age- and gender-matched healthy subjects were recruited from the University. Patients visited the hospitals for treatments and clinical evaluations. Blood samples were collected before treatment and at specific time points during treatment (weeks 0, 4, 12, 16, 52, 104, and 156; Fig. 1, Table 1). Dropouts included 1 subject in the SCIT group after 16 weeks of treatment because of poor compliance and 2 subjects who did not return for follow-up visits in the medication group after 52 weeks of treatment. Not all patients provided blood at every time point; the missing data from these patients were not included in the final analysis. Written informed consent was obtained from all adult patients, or parents of the children.

**Table 1:** The baseline (week 0) information; percentage of Treg, Th1 and Th2; and supernatant cytokine levels in PBMCs stimulated with a mixture of Der-p and serum in the SCIT, non-SCIT, and healthy subjects.

Variable	SCIT group		Non-SCIT group		Healthy group	
Patients, *n*^§^	20		20		20	
Gender (male/female), *n*^§^	13/7		14/6		10/10	
Age distribution (years)^§^	24.4 ± 1.5		19.5 ± 1.3		22.7 ± 1.4	
Patients, *n* (%)						
With AR only	4 (20.0)		3 (15.0)		–	
With asthma and AR	16 (80.0)		17 (85.0)		–	
SPT-diameter						
Der-p (mm)^§^	9.9 ± 0.7		9.1 ± 0.6		0.9 ± 0.0^†††,###^	
Der-f (mm)^§^	8.2 ± 0.6		8.3 ± 0.5		0.8 ± 0.0^†††,###^	
sIgE to Der-p (kU/L)^§^	73.2 ± 8.8		76.9 ± 8.4		0.2 ± 0.0^†††,###^	
sIgE to Der-f (kU/L)^§^	84.1 ± 9.5		89.4 ± 9.6		0.2 ± 0.0^†††,###^	
Total IgE (kU/L)^§^	665.7 ± 42.3		539.7 ± 40.8		49.6 ± 8.9^†††,###^	
SMS^§^	3.6 ± 0.1		4.0 ± 0.1		0.1 ± 0.0^†††,###^	
FEV_1_ (% predicted)^§^	90.7 ± 1.8		92.5 ± 1.7		95.1 ± 1.9	
EOS ( × 10^9^/L)^§^	0.5 ± 0.0		0.5 ± 0.0		0.1 ± 0.0^†††,###^	
	Unstimulated	Der-p Stimulated	Unstimulated	Der-p Stimulated	Unstimulated	Der-p Stimulated
**T cells (%)**						
Treg^§^	5.5 ± 1.9	6.3 ± 1.8	5.9 ± 2.2	5.3 ± 2.0	5.2 ± 1.7	6.1 ± 1.9
Th1^§^	13.4 ± 4.6	11.9 ± 5.0	10.8 ± 6.0	12.1 ± 6.4	14.3 ± 6.1	12.6 ± 6.8
Th2^§^	2.9 ± 1.6	2.4 ± 1.3	2.7 ± 1.7	2.5 ± 1.1	1.7 ± 1.3^†,#^	1.8 ± 1.4^†,#^
**Cytokines (pg/mL)**						
IL-4^§^	2.1 ± 0.4	3.2 ± 0.7 ∗	1.4 ± 0.3	2.3 ± 0.6 ∗	0.7 ± 0.1^†,#^	0.8 ± 0.1^††,##^
IL-5^§^	12.2 ± 5.4	1083.4 ± 236.3 ∗∗∗	11.4 ± 6.8	908.0 ± 360.6 ∗∗∗	9.8 ± 4.4	13.6 ± 5.7^†††,###^
IL-10^§^	18.2 ± 6.4	25.2 ± 29.4	15.5 ± 22.7	21.9 ± 26.2	27.9 ± 6.9	31.8 ± 5.0
IL-13^§^	38.7 ± 12.7	494.5 ± 87.5 ∗∗∗	28.1 ± 15.0	373.2 ± 90.4 ∗∗∗	32.7 ± 11.2	42.7 ± 15.3^†††,###^
IL-17^§^	17.0 ± 5.3	69.0 ± 19.6 ∗∗∗	12.7 ± 4.0	46.1 ± 8.2 ∗∗∗	9.1 ± 3.2	13.1 ± 5.7^†††,###^
IFN-γ^§^	42.2 ± 10.6	48.0 ± 14.0	25.4 ± 6.4	41.5 ± 8.4	21.5 ± 5.7	35.9 ± 7.2
TNF-α^§^	62.1 ± 26.4	316.3 ± 66.7 ∗∗∗	102.5 ± 53.7	360.1 ± 106.8 ∗∗∗	30.5 ± 8.1^†,#^	44.6 ± 17.0^†††,###^
TGF-β1^§^	2042.1 ± 241.4	2250.4 ± 246.1	1688.0 ± 235.0	1797.7 ± 224.3	1821.5 ± 307.2	2023.3 ± 315.6

Data are presented as mean ± SEM. §*P* > 0.05 when compared between the SCIT group and the non-SCIT group; †*P* < 0.05, ††*P* < 0.01, †††*P* < 0.001 compared with the SCIT group; #*P* < 0.05, ##*P* < 0.01, ###*P* < 0.001 compared with the non-SCIT group; ∗*P* < 0.05, ∗∗*P* < 0.01, ∗∗∗*P* < 0.001 compared with the unstimulated group. PBMC, peripheral blood mononuclear cell; SCIT, subcutaneous allergen immunotherapy; SPT, skin prick test; IgE, immunoglobulin E; sIgE, specific IgE; EOS, peripheral eosinophil counts; SMS, combined symptom and medication score; FEV_1_, forced expiratory volume in 1 s; Th, T helper cell; Treg, regulatory T cell; Der-p, *Dermatophagoides pteronyssinus*; Der-f, *Dermatophagoides farina*; IL, interleukin; IFN-γ, interferon-γ; TNF-α, tumor necrosis factor-α; TGF-β, transforming growth factor-β

### Skin prick tests

Sensitization to house dust mite aeroallergens, including Der-p, *Dermatophagoides farinae* (Der-f), and other common allergens (Soluprick SQ, ALKAbello A/S, Horsholm, Denmark), was assessed. A positive skin reaction was defined as a wheal size ≥3 mm after subtraction of the negative control.

### Detection of serum IgE and IgG4

The levels of total IgE and sIgE against Der-p, Der-f, and other common allergens such as dog dander in all serum samples were measured by a Pharmacia CAP fluorescence enzyme immunoassay system (ThermoFisher, Sweden) at week 0. The sIgE results are reported as kU/L, with a cut-off value of 0.35 kU/L and upper sIgE detection limit of 100 kU/L. Any sample with a sIgE level >100 kU/L was diluted and tested again. Serum Der-p sIgG4 levels were measured by a four-layer sandwich ELISA using methods that we had reported previously.^12^

### Spirometry and histamine bronchial provocation test

Forced vital capacity (FVC) and forced expiratory volume in 1 s (FEV_1_) were measured (weeks 0, 4, 12, 16, 52, 104, and 156) using a MicroQuark Spirometer (Cosmed), which met the standards of the American Thoracic Society and the European Respiratory Society.^13^ The findings are presented as percent predicted value (FEV_1_%).

### SCIT protocol

The patients were treated with subcutaneous injections of standardized aluminum-formulated Der-p Alutard-SQ vaccine (ALK-Abello A/S, Horsholm, Denmark). The treatment protocol followed the recommended updosing schedule of 16 weeks before reaching a maintenance dose of 100 000 Alutard-SQ given every 6–8 weeks, which was maintained to complete 3 years of SCIT.

### Clinical evaluations

The patients were requested to complete a symptom and medication diary during the whole course of treatment. They were asked to rate the symptoms of asthma (daytime: 0–5; night time: 0–4) and rhinitis (day or night time: 0–2) based on the severity and frequency of symptoms in disturbing daily activities and sleep.^14^ The medication score was calculated by assigning a score of 1–160 μg of budesonide or the equivalent dose of inhaled corticosteroid, or 130 μg of budesonide or the equivalent dose of nasal corticosteroid; each puff of salbutamol/terbutaline or the equivalent dose of other inhaled β_2_-agnoist; and 10 mg of oral loratadine or the equivalent dose of other oral anti-histamine. Combined symptom medication score (SMS) was defined as the sum of symptom scores and medication scores.^10^

### Assessment of T-lymphocyte subsets

For analysis of CD4^+^IL-4^−^IFN-γ^+^ Th1 and CD4^+^IL-4^+^IFN-γ^−^ Th2 cells, peripheral blood mononuclear cells (PBMCs) were isolated from peripheral blood using Ficoll-Paque and incubated with 25 μg/mL Der-p extract (ALK-Abello, Denmark) over a period of 72 h at 37 °C, during which 25 ng/mL phorbol 12-myristate 13-acetate (PMA), 1 μg/mL ionomycin, and 1.7 μg/mL monensine (all from Sigma, USA) were added for the final 4 h of the incubation period. At the end of incubation, the cells were stained with anti-CD4-PC5, *anti*-IL-4-PE, *anti*–IFN–γ-FITC, or isotype antibodies as control, and then assessed by flow cytometry (all antibodies and reagent from eBioscience, USA). For analysis of CD4^+^CD25^+^Foxp3^+^ Treg cells, PBMCs were incubated with 25 μg/mL Der-p extract over a period of 72 h at 37 °C. The cells were collected and stained with anti-CD4-FITC, anti-CD25-PC5, and *anti*-Foxp3-PE in accordance with the instruction manuals, detected by flow cytometry (Beckman Coulter Epics XL-MCL, USA), and analyzed using FCS Express software (version 4).

### Effector cell cytokine release inhibition assay

Serum inhibitory activity against cytokine release by Der-p-stimulated effector cells was detected by cytokine release inhibition assay (CRIA). Briefly, 10 μL of serum from SCIT and non-SCIT patients was incubated with 30 μL of Der-p allergen in a 96 well plate (the final concentration of Der-p was 0.15 μg/mL or 15 μg/mL, and the allergen concentration was referenced to the basophil activation test as described previously^1^) at 37 °C for 1 h. Subsequently, we added 200 μL of PBMCs (from 3 severe house dust mite (HDM)-sensitized donors, 2 × 10^6^ cells/mL) and incubated the samples at 37 °C for 24 h. After centrifugation, the supernatant was collected for cytokine determination. The supernatant concentrations of IL-4, IL-5, IL-10, IL-13, IL-17, IFN-γ, TNF-α, and TGF-β1 were tested by Bio-Plex Cytokine Assay (Bio-Rad, USA) in accordance with the manufacturer's instructions.

### Statistical analysis

Statistical analysis was performed using SPSS version 16.0 and GraphPad Prism 8.3.0. The Mann–Whitney *U* test was used to analyze between-group differences of the baseline data. A two-way ANOVA with Sidak's multiple comparisons test for two or more groups was used to analyze repeated measure data. Values are shown as mean ± SEM. Simple linear regression was employed to analyze the relationship between log Der-p sIgG4 levels, percentage of CD4^+^CD25^+^Foxp3^+^ Treg, CD4^+^IL-4^−^IFN-γ^+^ Th1, and CD4^+^IL-4^+^IFN-γ^−^ Th2 cells, and various cytokine levels. Differences were considered significant at values of *P* < 0.05.

## Results

### Patients’ characteristics and baseline levels of T-cell subsets

The baseline demographic data, SMS, antibody levels, and T-cell subsets levels of all subjects are shown in Table 1. There were no differences between the SCIT and non-SCIT groups in terms of gender, SMS, FEV_1_%, SPT-diameters, peripheral eosinophil counts, serum IgE levels, and percentage of CD4^+^CD25^+^Foxp3^+^ Treg and CD4^+^IL-4^−^IFN-γ^+^ Th1 cells (*P* > 0.05), while CD4^+^IL-4^+^IFN-γ^−^ Th2 levels were higher in SCIT and non-SCIT subjects than in healthy controls at baseline (*P* < 0.05). There were significantly higher levels of IL-4, IL-5, IL-13, IL-17, and TNF-α secretion from PBMCs upon Der-p stimulation in the allergic SCIT and non-SCIT groups (*P* < 0.05).

### Changes of clinical outcomes

Total symptom scores, Asthma symptom scores, Medication scores, and SMS decreased significantly after 52 weeks of treatment in SCIT patients compared with week 0 (*P* < 0.05). Rhinitis symptom scores decreased significantly after 104 weeks of treatment in SCIT patients compared with week 0 (*P* < 0.01). Total symptom scores, asthma symptom scores, rhinitis symptom scores, medication scores, and SMS in SCIT patients were significantly lower compared with non-SCIT patients at week 156 (*P* < 0.05), week 104 (*P* < 0.05), week 156 (*P* < 0.05), week 104 (*P* < 0.01), and week 52 (*P* < 0.05), respectively. FEV_1_% did not change significantly during treatment in either group (*P* > 0.05) (Supplementary Fig. 1A–F).

### Time course of treg, Th1, Th2, and serum sIgG4

**Fig. 2:**
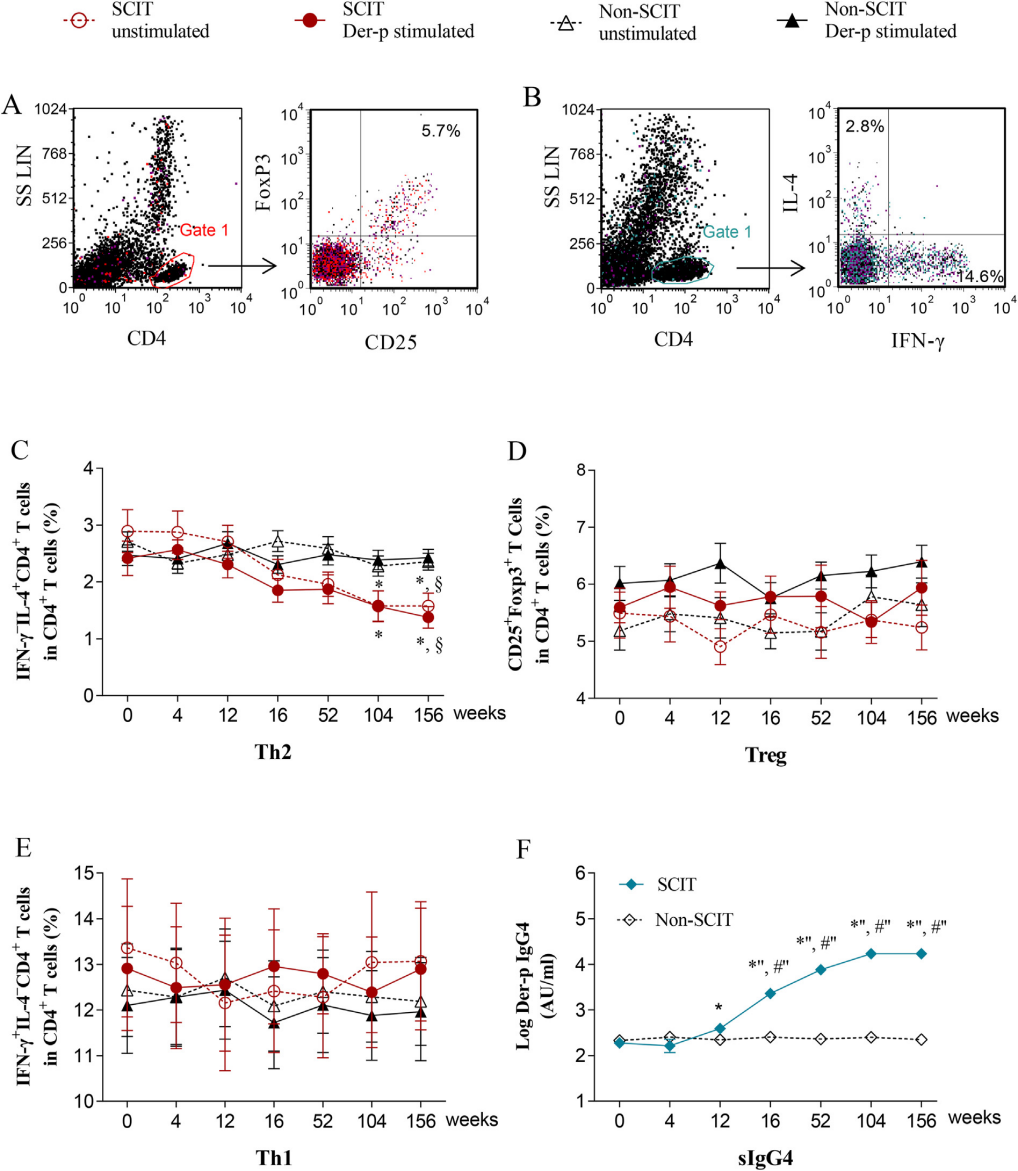
**Time course of the mean percentage of CD4^+^CD25^+^Foxp3^+^ Treg, CD4^+^IL-4^−^IFN-γ^+^ Th1, and CD4^+^IL-4^+^IFN-γ^−^ Th2 in the SCIT group and non-SCIT group**. Lymphocytes are identified by their scatter properties, i.e., the Treg, Th1 and Th2 cells are gated as CD4^+^CD25^+^Foxp3^+^ (A), CD4^+^IL-4^−^IFN-γ^+^ (B), and CD4^+^IL-4^+^IFN-γ^−^ (B), respectively. The mean percentage of Th2 (C), Treg (D), and Th1 (E) cells in CD4^+^ T cells from the SCIT group and non-SCIT group. Time course of Der-p sIgG4 (F) in SCIT group and non-SCIT group; the y-axis is a log scale. ∗*P* < 0.05, ∗' *P* < 0.01, ∗'' *P* < 0.001 when compared with week 0; #'' *P* < 0.001 when compared with non-SCIT group; §*P* < 0.05 compared with the non-SCIT group with or without Der-p stimulation. Foxp3, forkhead box protein 3; Th, T helper cell; Treg, regulatory T cell; IL, interleukin; IFN-γ, interferon-γ; Der-p, *Dermatophagoides pteronyssinus*; sIgG4, specific immunoglobulin G4.

Lymphocytes were identified by their scatter properties, and the Treg, Th1 and Th2 cells gated as SSC^low^CD4^+^CD25^+^Foxp3^+^ (Fig. 2A), SSC^low^CD4^+^IL-4^−^IFN-γ^+^ (Fig. 2B), and SSC^low^CD4^+^IL-4^+^IFN-γ^−^ (Fig. 2B), respectively. The percentage of Th2 cells in the SCIT group decreased significantly from week 104 to week 156 compared with week 0 both with and without Der-p stimulation (*P* < 0.05), and decreased significantly at week 156 both with and without Der-p stimulation compared with non-SCIT subjects with or without Der-p stimulation, respectively (*P* < 0.05, Fig. 2C). No significant changes were observed in the percentage of Treg and Th1 cells during the time course of treatment compared with week 0 in SCIT and non-SCIT patients (*P* > 0.05, Fig. 2D and E). The levels of Der-p sIgG4 in SCIT patients significantly increased starting from week 12 compared with week 0 (*P* < 0.05), and significantly increased from weeks 16 to week 156 compared with non-SCIT subjects (*P* < 0.01, Fig. 2F).

### Inhibition of effector cell cytokine release using serum samples of SCIT and non-SCIT patients

**Fig. 3:**
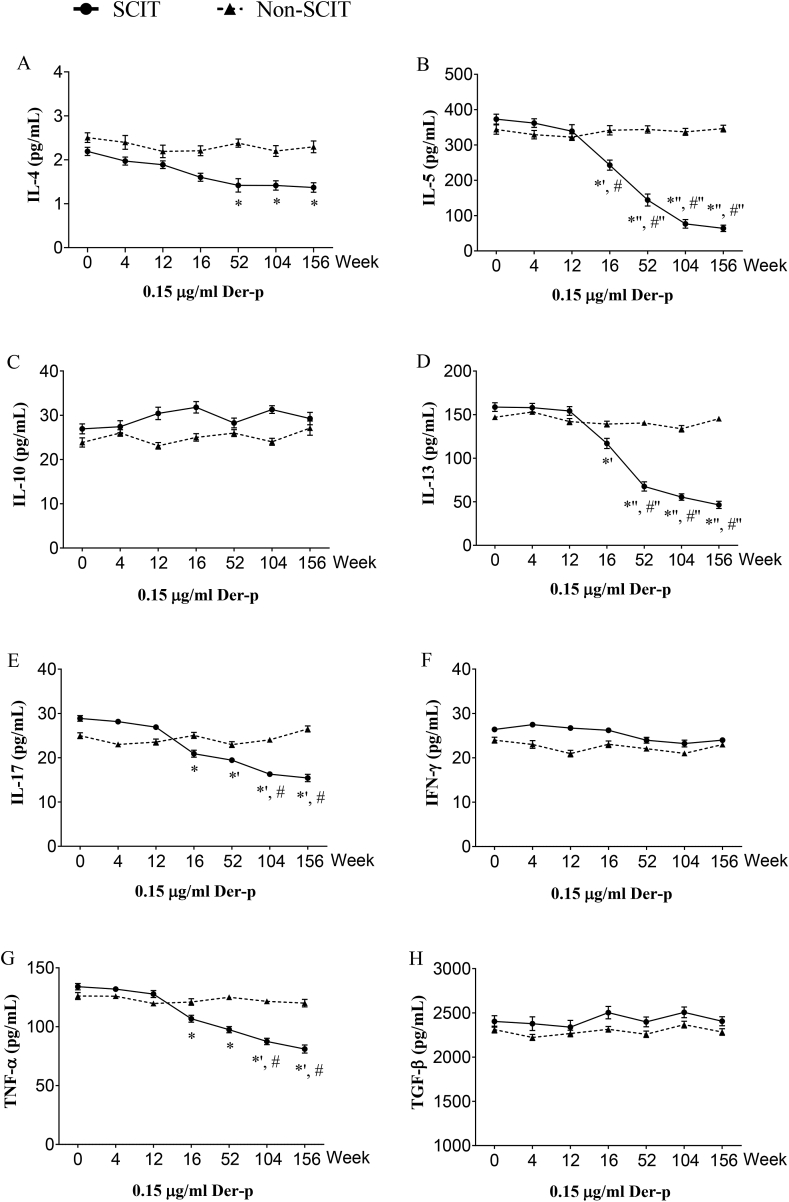
**Time course of the inhibition of cytokine release at submaximal allergen concentration**. The effector cell cytokine release inhibition assay was performed with serum from the SCIT group and non-SCIT group incubated with 0.15 μg/mL Der-p allergen. ∗*P* < 0.05, ∗' *P* < 0.01, ∗'' *P* < 0.001 when compared with week 0; #*P* < 0.05, #' *P* < 0.01, and #'' *P* < 0.001 when compared with non-SCIT group; Der-p, *Dermatophagoides pteronyssinus*; IL, interleukin; AIT, allergen immunotherapy; IFN-γ, interferon-γ; TNF-α, tumor necrosis factor-α; TGF-β1, transforming growth factor-β1.

The levels of supernatant cytokines were not changed when stimulated with 15 μg/mL of Der-p allergen and serum from SCIT and non-SCIT patients (Supplementary Figure 2). However, when stimulated with 0.15 μg/mL of Der-p allergen, as compared with week 0, the levels of cytokines in supernatants of HDM-sensitized PBMCs and serum from SCIT patients decreased significantly after 16 weeks for IL-5, IL-13, IL-17, and TNF-α (*P* < 0.05), after 52 weeks for IL-4 (*P* < 0.05), but not for IL-10, IFN-γ, and TGF-β1, and not for serum from non-SCIT patients (*P* > 0.05, Fig. 3). As compared with non-SCIT subjects, serum from SCIT patients led to significant lower release after 16 weeks for IL-5 (*P* < 0.05), after 52 weeks for IL-13 (*P* < 0.001), after 104 weeks for IL-17, and TNF-α from PBMC (*P* < 0.05, Fig. 3).

### The relationship between levels of cytokines and der-p sIgG4 and T-cell subsets

**Fig. 4:**
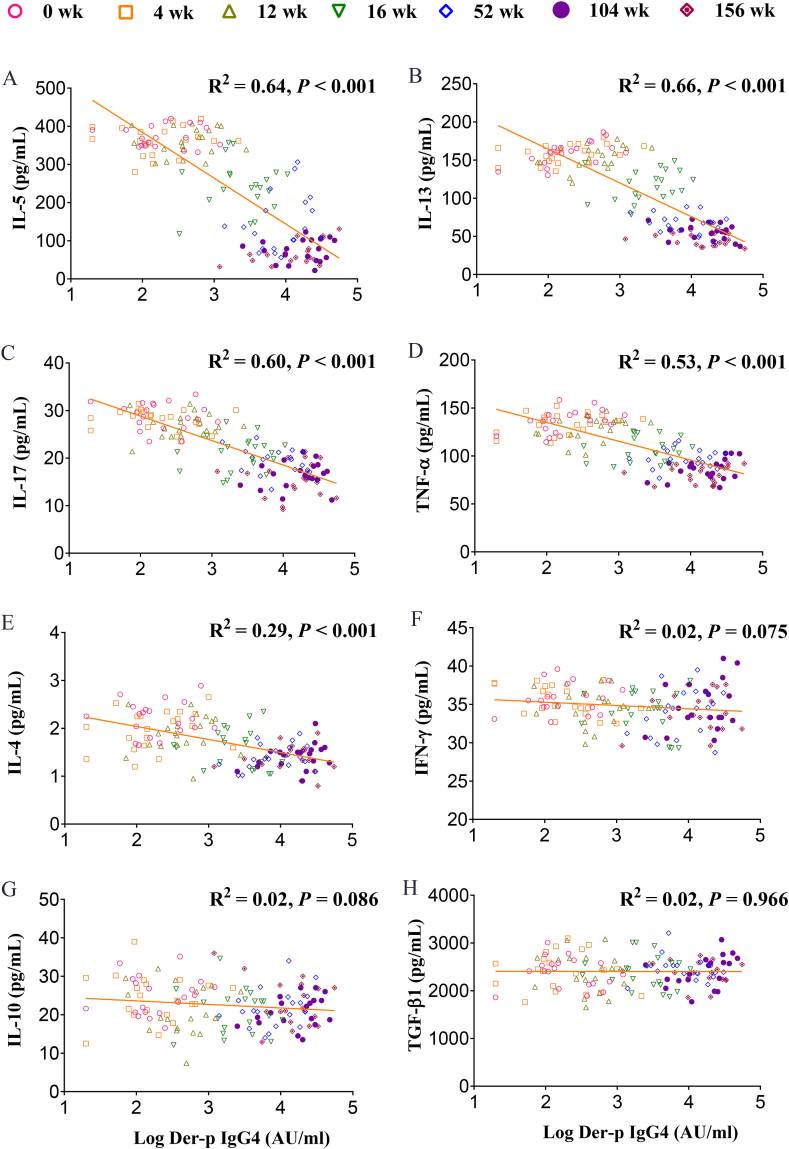
**Linear regression between Der-p sIgG4 and submaximal allergen concentration stimulated cytokine release inhibition assay in the SCIT group at all time points during the treatment**. IL-5 (A), IL-13 (B), IL-17 (C), TNF-α (D), IL-4 (E), IFN-γ (F), IL-10 (G), and TGF-β1 (H). ∗∗∗*P* < 0.001, linear regression. Der-p, *Dermatophagoides pteronyssinus*; IL, interleukin; IFN-γ, interferon-γ; TNF-α, tumor necrosis factor-α; TGF-β1, transforming growth factor-β1.

The levels of Der-p sIgG4 had significant negative linear associations with supernatant IL-5, IL-13, IL-17, TNF-α, and IL-4 in sensitized PBMCs stimulated with 0.15 μg/mL of Der-p allergen and serum of SCIT subjects at all time points after the treatment (IL-5, R^2^ = 0.64, *P* < 0.001; IL-13, R^2^ = 0.66, *P* < 0.001; IL-17, R^2^ = 0.60, *P* < 0.001; TNF-α, R^2^ = 0.53, *P* < 0.001; IL-4, R^2^ = 0.29, *P* < 0.001; IFN-γ, R^2^ = 0.02, *P* > 0.05; IL-10, R^2^ = 0.02, *P* > 0.05; and TGF-β1, R^2^ = 0.00, *P* > 0.05; Fig. 4). The above correlations were not found in non-SCIT subjects (Supplementary Figure 3).

## Discussion

In this study, we found that Der-p SCIT resulted in significant improvement in controlling asthma/rhinitis symptoms as well as reduction in medication requirements starting after 12 weeks of SCIT. The percentage of Th2 cells in SCIT patients significantly decreased from weeks 104–156 compared with baseline. Serum obtained from SCIT patients significantly blocked cytokine release from effector cells, and a significant inverse relationship was demonstrated between the levels of Der-p sIgG4 and effector cell cytokine release.

Due to ethical and practical considerations, it was impracticable to perform a double-blind, placebo-controlled study in a real-world setting as we demonstrated previously^14^ in this three-year clinical observation. The current investigation confirmed that AIT is an effective treatment for allergic disease, which is consistent with our previous studies^12^, ^14^ and other studies.^15^, ^16^ Improved compliance associated with study participation in AIT study may also improve efficacy in both the AIT and the placebo group,^14^ this may explain the drop in symptom and medication scores seen in all patients. Multi-center, large sample size, real world study is needed for further investigation on the biologic effect in responses to AIT. The mechanism of AIT involved the shift from a Th2 to a Th1 cell-dominated immune response. It has been demonstrated that the proliferative response of peripheral blood T cells to allergens is reduced after immunotherapy with venom or house dust mite,^17^ and that a shift from Th2 to Th1 milieu occurs in grass pollen immunotherapy.^3^^,^^18^ In this study, we found that AIT could downregulate the frequency of Th2 cells, which was not accompanied with an increase in Th1 cell number. In contrast, similar changes in Th2 cells were not observed in other studies after AIT.^19^, ^20^, ^21^ Wei et al. demonstrated that the frequency of allergen-specific Th2 cells did not decrease significantly after immunotherapy.^22^ The controversial results about the change in Th2 cells during AIT may be related to the duration and administration type of AIT. Besides, other study found that Th2 subpopulations such as Th2A and CD38^+^ Th2A subsets may serve as valuable biomarkers in the adaptive responses during AIT.^23^ Overall, the variability of Th1/Th2 immune deviation in the peripheral circulation after immunotherapy may not sufficiently reflect the clinical features of immunotherapy.^24^

AIT-induced allergen tolerance is also associated with the generation of natural Treg (nTreg), which is characterized by expression of Foxp3 at the early stage of AIT.^18^ Foxp3 is critical for the function of the nTreg and represents their specific marker.^25^ Radulovic et al^26^ found that the number of CD4^+^Foxp3^+^ Treg cells in the nasal mucosa increased after grass subcutaneous-injection immunotherapy and correlated with clinical efficacy, which supported the induction of immunosuppression of Treg cells in AIT immune tolerance. Similarly, Lin et al^20^ demonstrated that AIT could induce the expression of Foxp3 and upregulate the functional activity of nTreg cells. In contrast, other studies^19^^,^^22^ found that the percentage of peripheral Foxp3^+^ Treg cells did not change after 1 year of house dust mite immunotherapy. In the current study, the frequency of CD4^+^CD25^+^Foxp3^+^ Treg cells in the SCIT group did not significantly change during the treatment. In general, AIT may preferentially induce Treg cells in the local tissue rather than in peripheral blood, and different markers, such as CD4^+^Foxp3^+^ Treg or CD4^+^CD25^+^Foxp3^+^ Treg, used in the studies may also have yielded conflicting data. Moreover, some studies suggested that the inducible IL-10-producing type 1 regulatory (Tr1) cells are more critical than nTreg in the regulation of humoral immune response.^4^ Those studies found that IL-10 and TGF-β secreted by Tr1 may switch allergen-specific B-cells to induce IgG4 instead of sIgE.^4^

This current study showed that the baseline level of Th2 cytokines (IL-4, IL-5, IL-13, IL-17, and TNF-α) in SCIT and non-SCIT subjects increased significantly after Der-p stimulation, confirming that cross-linking of IgE receptors on the surface of effector cells by IgE-allergen complexes could trigger an immediate-type immune reaction and release Th2 cell cytokines in allergic patients.^27^ IL-10 may also have other functions besides inducing IgG antibodies, such as inhibition of T cell proliferation and cytokine production,^28^ induction of T cell anergy,^29^ and induction of mast cell apoptosis.^30^ TGF-β1 is an important immunosuppressive cytokine produced by many cell types including Treg cells.^31^ The change in IL-10 and TGF-β1 in patients receiving AIT is controversial. Studies have shown that AIT could induce allergen-specific IL-10-secreting cells at the early stage of immunotherapy.^32^, ^33^ Wei et al.^24^, ^34^ showed that the level of IL-10 in the supernatant of PBMCs was significantly increased after AIT. In contrast, another study^35^ found no significant change in IL-10 secreted by allergen-specific PBMCs after 8 weeks of AIT. As for TGF-β, Jutel et al.^32^ found that the TGF-β was increased after AIT, while another study^36^ reported that the level of TGF-β in the supernatant of PBMCs was unchanged. These inconsistent results may reflect that the production of cytokines in the PBMC supernatant involves a complex network of immune effector cells, especially mast cells and eosinophils, and may also relate to the allergens used in AIT, the duration and administration type of AIT, and the status of atopic disease.

We found that AIT could induce a substantial increase in Der-p sIgG4, which is consistent with our previous findings and those of other studies, confirming that sIgG4 is a prominent immunological change induced by AIT.^6^ The magnitude of increase in sIgG4 concentrations may be related to the concentration of the allergen^37^ and the duration of AIT treatment.^12^ IgG4 is a unique antibody involved in a continuous process of heavy and attached light-chain exchange. This process, which is referred to as “Fab-arm exchange”, results in monovalent and non-crosslinking antibodies; in other words, the binding of IgG4 and allergens could reduce the free allergen concentration but does not induce effector cell activation.^38^ The allergen-specific IgG4 could be measured in allergic non-AIT patients for the natural exposure; however, no blocking activity was detected. The IgE blocking activity may be due to high IgG4 concentrations or high IgG4 affinity after prolonged immunization.^39^ Specific IgG4 has been proposed to compete with sIgE to form the allergen-IgE complex, thereby inhibiting complex binding to B cells^36^^,^^40^ and preventing allergen-dependent T cells activation.^41^ It is also associated with the inhibition of allergen-induced effector cell activation^42^^,^^43^ and reduced allergen sensitivity.^44^ Our previous basophil activation test inhibition experiments showed that AIT-induced serum inhibiting antibodies reduced basophil allergen threshold sensitivity, but had no effect on basophil reactivity.^1^ In our current cytokine release inhibition experiments, we found that serum obtained from SCIT patients inhibited IL-4, IL-5, IL-13, IL-17, and TNF-α release from effector cells at submaximal allergen concentration stimulation and had a significant inverse correlation between the levels of Der-p sIgG4 and cytokine release. However, IL-10, IFN-γ, and TGF-β1 did not change significantly during the treatment, these cytokines may not be sensitive to allergen stimulation. Obtaining suitable PBMCs from HDM-sensitized patients is an important procedure for the cytokine release inhibition experiment. It is sensitive to allergen stimulation when the PBMCs contain high levels of cytokines, especially IL-5 and IL-13. The experiment is also repeatable when the PBMCs are obtained from the same individual. However, the cytokines in PBMCs have individual variances, and the secretion patterns of released cytokines may be different when the PBMCs are obtained from different patients. In general, the cytokine release inhibition experiment represents a reproducible way for the detection of antibodies inhibiting allergens to cross-link IgE. Besides being regulated by an IL-10 and TGF-β paracrine mechanism, the suppression mechanism of AIT may also involve with induction of IL-10 and TGF-β expression on the cell-surface of Treg as a modulator activate of Treg and interact with the target lymphocytes.^22^, ^25^ Furthermore, serum obtained from AIT patients could not inhibit effector cell cytokine release at maximal allergen concentration stimulation, which may suggest that SCIT cannot reduce the reactivity of anaphylactic cells in PBMCs. Thus, IgG4 antibodies may play an important role in reducing the sensitivity of effector cell cytokine production during AIT, and the function of inhibition ability increases along with increasing sIgG4. In addition, other antibodies, such as IgA, IgG1, and IgG2, might also contribute to the inhibition associations. Study found that specific IgG2 increased after house dust mite AIT, and have significant correlation between IgG2 and IgE in high clinical benefit response individuals.^45^ Additional studies, with purified allergen-specific IgG antibodies, IgE, and effector cells are needed to clarify the interrelationship between these cellular responses and humoral allergen-specific IgG and IgE antibodies. Allergic adverse events may occur during AIT with the vaccines prepared from whole allergen. Purified allergen component or peptide AIT may reduce capacity to cross-link IgE and activate mast cells and basophils,^46^^,^^47^ the precise mechanism needs further investigation. There were several limitations in this study. First, we performed experiments using serum from patients who had received SCIT, and other antibodies, such as IgA and IgG1, may also contribute to inhibitory associations. Second, we recruited children and adults in this study, and children were more likely to produce higher levels of sIgG4 during the shorter AIT period compared to adults.^12^ Additional studies, particularly those with larger sample sizes and purified sIgG4 from serum, are needed to confirm our preliminary findings.

In conclusion, AIT is an effective treatment for allergic diseases. The mechanisms of AIT involve both the cellular and humoral immune responses during allergen immunotherapy. AIT could reduce the percentage of Th2 cells and massively induce production of allergen-specific IgG4 antibodies. SCIT-related reduction in cytokine release from effector cells in Der-p allergic rhinitis and/or asthma patients may be involved with induction of sIgG4.

## Abbreviations

AIT; allergen immunotherapy, CRIA; cytokine release inhibition assay, Der-f; *Dermatophagoides farinae*, Der-p; *Dermatophagoides pteronyssinus*, FEV_1_; forced expiratory volume in 1 s, Foxp3; forkhead box protein 3, FVC; forced vital capacity, HDM; house dust mite, IL; interleukin, PBMC; peripheral blood mononuclear cell, SCIT; subcutaneous allergen immunotherapy, sIgE; specific immunoglobulin E, sIgG4; specific immunoglobulin G4, SMS; combined symptom medication score, SPT; skin prick test, TGF-β; transforming growth factor-β, Th; T helper cell, TNF; tumor necrosis factor, Treg; regulatory T cell

## Funding

This study was funded by 10.13039/501100001809National Natural Science Foundation of China, China (U1801286, 8211101139), and Science and Technology Program of Guangzhou (202102010011), Zhongnanshan Medical Foundation of Guangdong Province (ZNSA-2020003), Zhongnanshan Medical Foundation of Guangdong Province (ZNSA-2020013), Guangdong Provincial Scientific Project (2017B020226006) and Open Project of State Key Laboratory of Respiratory Disease, China (SKLRD-OP-202004) to J. L., and supported by the 10.13039/501100001809National Natural Science Foundation of China [81500024] to Mulin Feng.

## Ethics statements

This study was approved by the Ethics Committee of the First Affiliated Hospital of Guangzhou Medical University and registered in the Chinese Clinical Trial Registry (ChiCTR–OOC–15006207).

## Author contributions

J.L. and M.L.F. designed the study, collected the data, and performed the statistical analysis. Q.J.S. and N·N.R. conducted the experiments, performed the statistical analysis, and drafted the manuscript. X.N.Z. and Y.D. were involved in the statistical analysis. M.X., X.S., T.L. and G.L. were involved in the data collection.

## Consent for publication

All authors have read and approved to submit this paper to the journal for consideration of publication.

## Data availability statement

The data that support the findings of this study are available from the corresponding author upon reasonable request.

## Declaration of competing interest

The authors declare no conflict of interest.

## Appendix A Supplementary data

Supplement Fig. 1 SMS and FEV1% over time. Time course of mean SMS (A) and FEV_1_% predicted (B) in SCIT group and non-SCIT group. ∗*P* < 0.05, ∗' *P* < 0.01, ∗'' *P* < 0.001 when compared with week 0; #*P* < 0.05, #' *P* < 0.01, #'' *P* < 0.001 when compared with the non-SCIT group. SMS, combined symptom and medication score; FEV_1_, forced expiratory volume in 1 s; SCIT, subcutaneous allergen immunotherapy. Supplement Fig. 1

Supplement Fig. 2 Time course of the inhibition of cytokine release at maximal allergen concentration. The effector cell cytokine release inhibition assay was performed with serum from SCIT group and non-SCIT group incubated with 15 μg/mL Der-p allergen. Not significant compared with week 0. Not significant compared with the non-SCIT group. Der-p, Dermatophagoides pteronyssinus; IL, interleukin; IFN-γ, interferon-γ; TNF-α, tumor necrosis factor-α; TGF-β1, transforming growth factor-β1. Supplement Fig. 2

Supplement Fig. 3 Linear regression between Der-p sIgG4 and submaximal allergen concentration in the non-SCIT group at all time points during treatment in the cytokine release inhibition assay. IL-5 (A), IL-13 (B), IL-17 (C), TNF-α (D), IL-4 (E), IFN-γ (F), IL-10 (G), and TGF-β1 (H). Der-p, Dermatophagoides pteronyssinus; IL, interleukin; IFN-γ, interferon-γ; TNF-α, tumor necrosis factor-α; TGF-β1, transforming growth factor-β1. Supplement Fig. 3
