# Supplementary figures and images for: Specific immunoglobulin G4 correlates with Th2 cytokine reduction in patients with allergic asthma treated by Dermatophagoides pteronyssinus subcutaneous immunotherapy
Source: World Allergy Organ J. 2023 Jan 26;16(1):100715. doi: 10.1016/j.waojou.2022.100715 (PMC9937843; doi:10.1016/j.waojou.2022.100715)

Supplemental figure 1

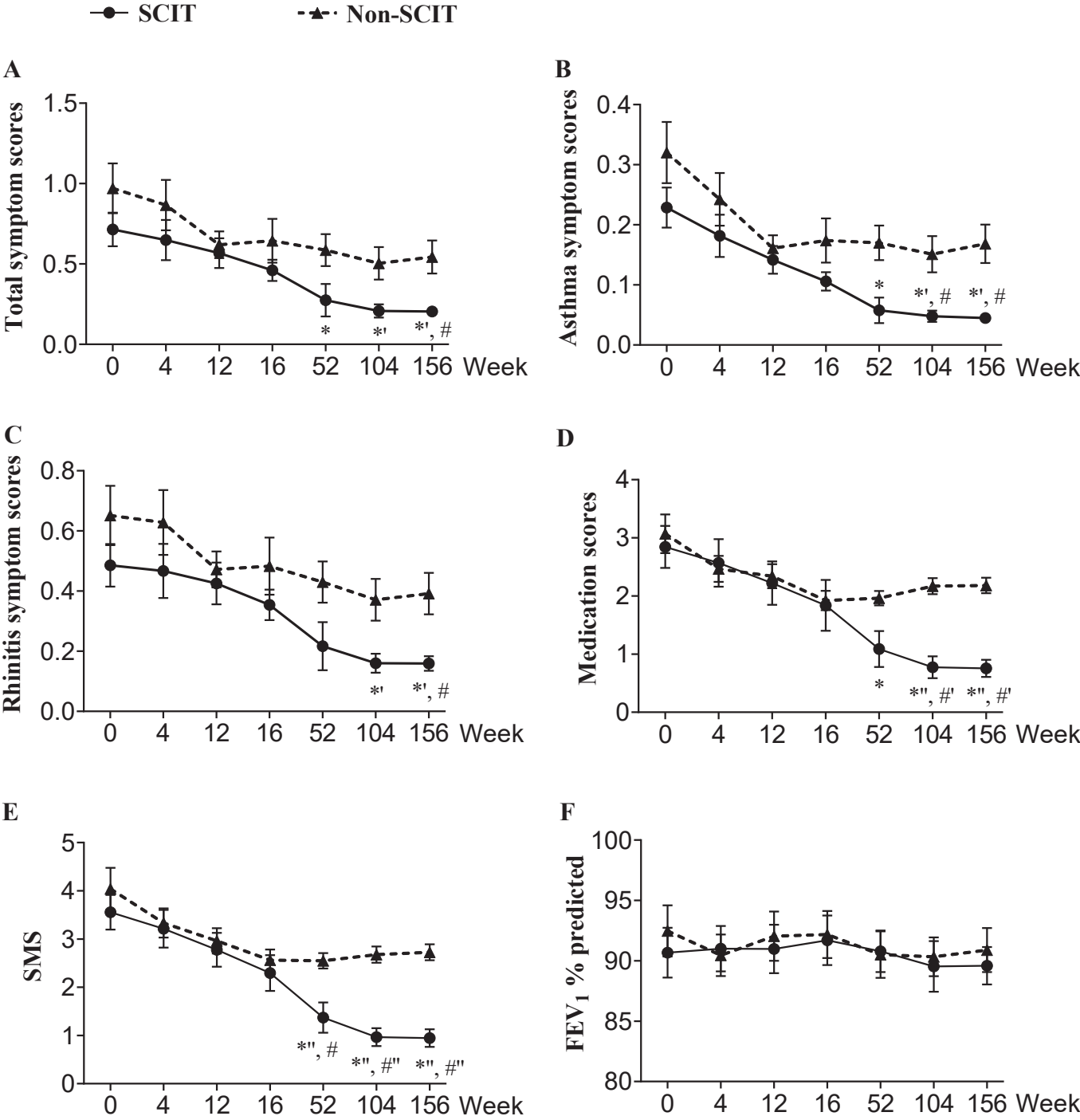

Supplement: Supplement Fig. 1 — SMS and FEV1% over time. Time course of mean SMS (A) and FEV1% predicted (B) in SCIT group and non-SCIT group. ∗P < 0.05, ∗' P < 0.01, ∗'' P < 0.001 when compared with week 0; #P < 0.05, #' P < 0.01, #'' P < 0.001 when compared with the non-SCIT group. SMS, combined symptom and medication score; FEV1, forced expiratory volume in 1 s; SCIT, subcutaneous allergen immunotherapy. [file mmc1.pdf]

Supplemental figure 2

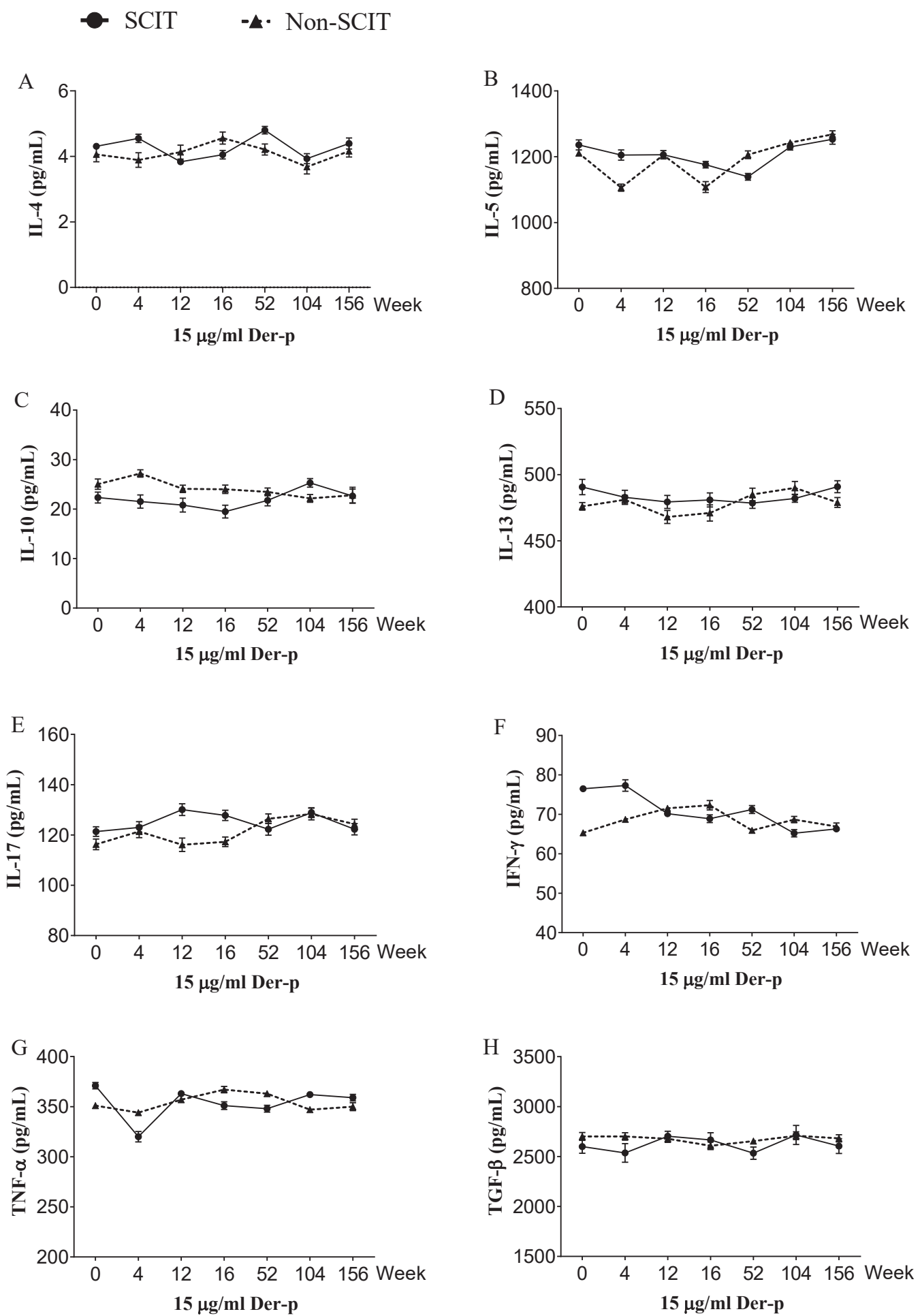

Supplement: Supplement Fig. 2 — Time course of the inhibition of cytokine release at maximal allergen concentration. The effector cell cytokine release inhibition assay was performed with serum from SCIT group and non-SCIT group incubated with 15 μg/mL Der-p allergen. Not significant compared with week 0. Not significant compared with the non-SCIT group. Der-p, Dermatophagoides pteronyssinus; IL, interleukin; IFN-γ, interferon-γ; TNF-α, tumor necrosis factor-α; TGF-β1, transforming growth factor-β1. [file mmc2.pdf]

# Supplement figure 3

○ 0 wk    □ 4 wk    △ 12 wk    ▽ 16 wk    ◇ 52 wk    ● 104 wk    ◆ 156 wk

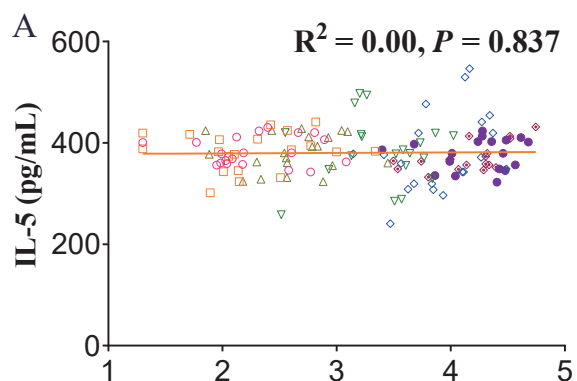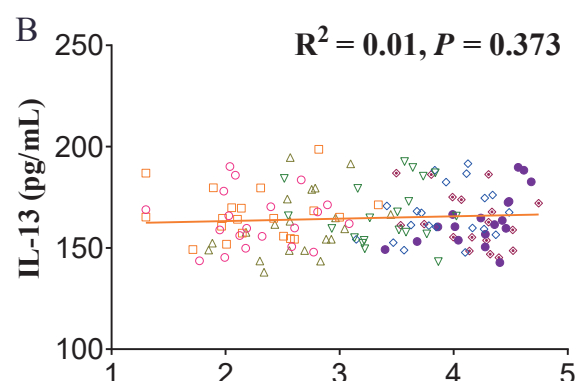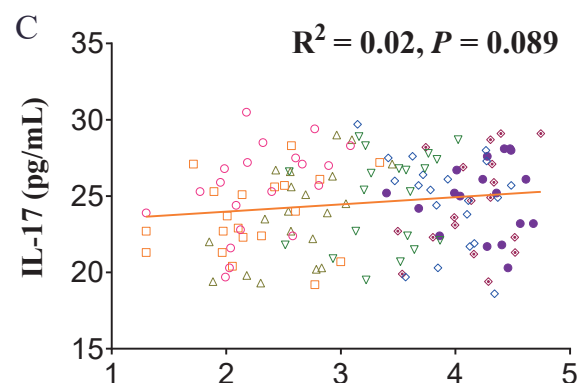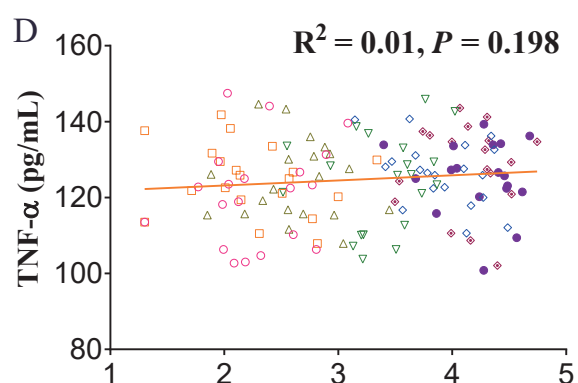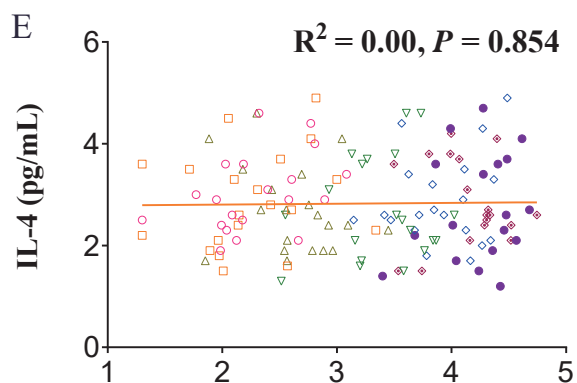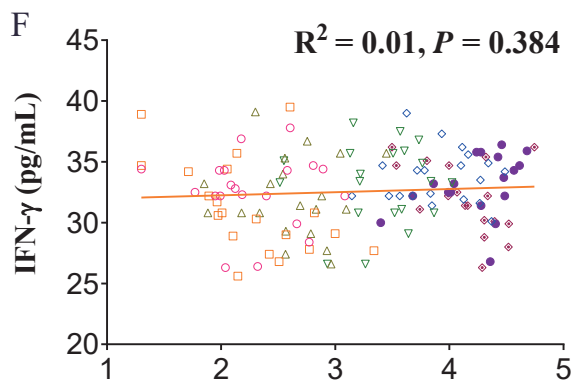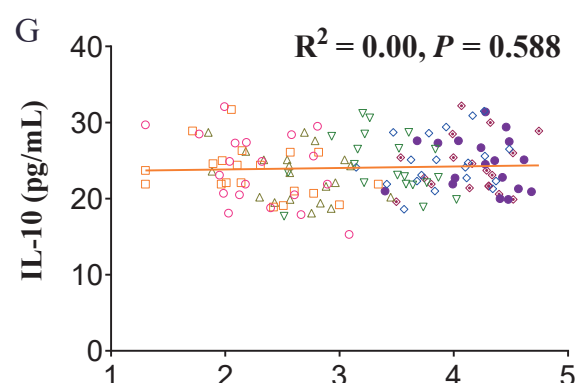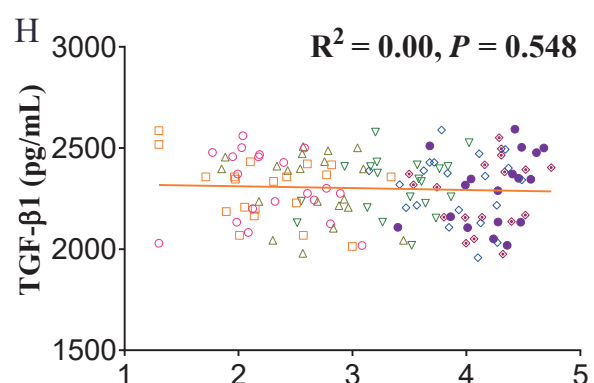

Log Der-p IgG4 (AU/ml)

Log Der-p IgG4 (AU/ml)

Supplement: Supplement Fig. 3 — Linear regression between Der-p sIgG4 and submaximal allergen concentration in the non-SCIT group at all time points during treatment in the cytokine release inhibition assay. IL-5 (A), IL-13 (B), IL-17 (C), TNF-α (D), IL-4 (E), IFN-γ (F), IL-10 (G), and TGF-β1 (H). Der-p, Dermatophagoides pteronyssinus; IL, interleukin; IFN-γ, interferon-γ; TNF-α, tumor necrosis factor-α; TGF-β1, transforming growth factor-β1. [file mmc3.pdf]
